# Germline mosaicism in *TCF20*-associated neurodevelopmental disorders: a case study and literature review

**DOI:** 10.1038/s10038-025-01323-3

**Published:** 2025-02-26

**Authors:** Jessie Poquérusse, Whitney Whitford, Juliet Taylor, Nerine Gregersen, Donald R. Love, Bobby Tsang, Kylie M. Drake, Russell G. Snell, Klaus Lehnert, Jessie C. Jacobsen

**Affiliations:** 1 School of Biological Sciences, The University of Auckland, Auckland, New Zealand; 2 Centre for Brain Research, The University of Auckland, Auckland, New Zealand; 3 Genetic Health Service New Zealand, Auckland City Hospital, Auckland, New Zealand; 4 Diagnostic Genetics, LabPLUS, Auckland City Hospital, Auckland, New Zealand; 5 Pediatrics and Newborn Services, Waitakere Hospital, Auckland, New Zealand; 6 Canterbury Health Laboratories, Christchurch, New Zealand; 7 Present Address: Genetic Pathology, Sidra Medicine, Doha, Qatar

**Keywords:** Autism spectrum disorders, Genetic testing

## Abstract

Autosomal dominant variants in transcription factor 20 (*TCF20*) can result in *TCF20*-associated neurodevelopmental disorder (TAND), a condition characterized by developmental delay and intellectual disability, autism, dysmorphisms, dystonia, and variable other neurological features. To date, a total of 91 individuals with TAND have been reported; ~67% of cases arose de novo, while ~10% were inherited, and, intriguingly, ~8% were either confirmed or suspected to have arisen via germline mosaicism. Here, we describe two siblings with a developmental condition characterized by intellectual disability, autism, a circadian rhythm sleep disorder, and attention deficit hyperactivity disorder (ADHD) caused by a novel heterozygous single nucleotide deletion in the *TCF20* gene, NM_001378418.1:c.4737del; NP_001365347.1:p.Lys1579Asnfs*36 (GRCh38/hg38). The variant was not detected in DNA extracted from peripheral blood in either parent by Sanger sequencing of PCR-generated amplicons, or by deep sequencing of PCR amplicons using MiSeq and MinION. However, droplet digital PCR (ddPCR) of DNA derived from early morning urine detected the variation in 3.2% of the father’s urothelial cells, confirming germline mosaicism. This report is only the second to confirm with physical evidence *TCF20* germline mosaicism and discusses germline mosaicism as a likely under-detected mode of inheritance in neurodevelopmental conditions.

## Introduction

Transcription factor 20 (*TCF20, MIM 603107*) has emerged as a syndromic neurodevelopmental disorder (NDD)-associated gene [1–4]. *TCF20* is expressed in human brain pre- and early postnatally, reaching peak developmental expression between 7 weeks post-conception and 2 years of age [5]. *TCF20* is located in the distal region of chromosome 22 (22q13.2) which is involved in human neurodevelopmental processes, and is predicted to be highly intolerant to loss of function (LOF) variants (pLI = 1) [6]. It is ubiquitously expressed through adulthood, and highly expressed in the cerebellum [7].

Animal models have shown that *TCF20* is highly expressed in the pre-migratory neural crest cells of chicken embryos [8], and in the hippocampus and cerebellum of mice during brain development [9]. The TCF20 protein, also named AR1, SPBP, or SPRE-binding protein, acts as a transcriptional activator which localizes to the nucleus and binds and controls the regulatory region of the gene encoding the extracellular matrix breakdown protein metalloproteinase 3 (MPP3)/stromelysin [10]. It has been found to also upregulate expression of a number of transcription factors, such as JUN, SP1, PAX6 and ETS1 [11, 12]. Recent functional work in mice has shown that *TCF20* is essential for neurogenesis, promoting the expression of *TDG*, which controls DNA methylation at the T‐cell factor 4 (TCF-4) promoter, thereby regulating its expression and modulating neural differentiation, deficits in which are associated with autism [13].

In humans, variants in the *TCF20* gene can result in developmental and intellectual disability, autism, dysmorphisms, and neurological features, alongside various other impairments spanning gastrointestinal issues, hepatic issues, and skeletomuscular systems. Pathogenic variants in *TCF20* act in an autosomal dominant mode (MIM 618430; Orphanet 35099) and result in *TCF20*-associated neurodevelopmental disorders (TAND) [14].

The *TCF20* and *RAI* (retinoic acid-induced 1) genes have likely evolved from a common ancestor by genome duplication [15]. Deletions or loss of function variants in *RA1* result in Smith-Magenis syndrome (MIM 182290), while duplications in *RA1* result in Potocki-Lupski syndrome (MIM 610883), both characterized by a similar constellation of mental disability and various congenital craniofacial and skeletal dysmorphisms.

To date, this case report and nine other publications [3, 16–23] including four large cohort studies [14, 24–26], have identified 76 unique pathogenic variants in or affecting (e.g. by chromoanagenesis) the *TCF20* gene in 91 individuals from 83 different families (Fig. 3 and Supplementary Table 1). Among these, Babbs et al. and Torti et al. described cases of a likely germline mosaic nature [16, 26], while only Schneeweiss et al. confirmed germline mosaic inheritance, of maternal origin, of a *TCF20* variant in an affected sibling [21].

Here, we report two affected siblings with developmental and intellectual disability, autism, dysmorphic features, and neurological variances. Whole exome sequencing (WES) of peripheral blood-derived DNA from the eldest sibling (II:1) identified a previously unreported *TCF20* frameshift variant, NC_000022.11:g.42210575del; NM_001378418.1:c.4737del; NP_001365347.1:p.Lys1579Asnfs*36 (GRCh38/hg38; MANE Select transcripts). The variant was also identified in peripheral blood-derived DNA in the younger sibling (II:2), but not identified in the parents’ peripheral blood by Sanger sequencing of PCR-generated amplicons. It was, however, detected by droplet digital PCR (ddPCR) in 3.2% of the father’s urothelial cells from an early morning urine sample.

This case report is only the second confirmed case with physical evidence of germline mosaicism in *TCF20*-associated neurodevelopmental disorders and the first to confirm the inheritance of the same *TCF20* variant in siblings. Our findings suggest germline mosaicism could be an under-detected mode of transmission for neurodevelopmental conditions with important implications for genetic counseling.

## Materials and methods

### Clinical report

#### II:1

The proband was the first child of a healthy Cook Island mother and Samoan father. She was born in New Zealand after a normal pregnancy at 40 weeks gestation. She experienced early post-natal global developmental delay. She was seen in the clinic for mild global development delay at 2 years and 4 months of age and met formal criteria for autism with intellectual disability. On examination at age 15, she was found to display immature speech, using short phrases and pointing to attract attention, while making reasonable eye contact. She has no obvious stereotypies or sensory processing issues. She has a circadian rhythm sleep disorder which is successfully controlled with melatonin and suffers from attention hyperactivity attention disorder (ADHD) which is successfully treated with methylphenidate. She experiences gastrointestinal distress in the form of intermittent constipation, and has mild left-hand side weakness and joint hypermobility. She has dysmorphisms in the form of posteriorly rotated small ears with small ear lobes. She has single palmar creases in the right hand and three café-au-lait macules. A brain MRI scan at age 3 years revealed no abnormal findings.

#### II:2

The proband’s younger brother was also New Zealand-born at 40 weeks gestation. He experienced early post-natal global developmental delay, intellectual disability, and autism. At 4.5 years of age, he was found to have left facial weakness with generalized hypotonia. He also suffers from a developmental coordination disorder. At 5.5 years of age, he was found to have symptoms of ADHD and dyspraxia, leading to a formal diagnosis of ADHD. He also has obesity and right exotropia. He is non-dysmorphic.

### DNA extraction and whole exome sequencing

High quality genomic DNA for II:1 was extracted from whole blood cells using the Qiagen Gentra Puregene Blood Kit according to manufacturer’s instructions, after which the DNA was quantified by Nanodrop, Qubit, and agarose gel electrophoresis.

Whole exome sequencing (WES) was carried out using the SureSelect XT Human All Exon v5 (Agilent, Santa Clara, CA, USA) and Illumina HiSeq 2000/2500 (Otogenetics Corporation, Atlanta, GA, USA) to generate 100 bp paired-end reads at an average read depth of 48x. Sequence alignment and variant calling for single nucleotide variations (SNVs) and insertions and deletions (indels) of WES data was carried out as previously described, but converted to GRCh38/hg38 coordinates using UCSC’s LiftOver tool [27]. WES alignments were viewed using the Integrated Genomics Viewer (IGV, v2.3.55) [28].

### WES analysis

A combined total of 93,403 high-confidence variants were identified in exonic and splice-junction regions across individual II:1’s whole exome sequence. Variants were first removed if observed at a minor allele frequency (MAF) > 0.01 in healthy populations from the ExAC database [29], or at a MAF > 0.05 in unrelated cases from our in-house database. This yielded a total of 4548 variants. Variants were filtered such as to only keep homozygous, compound heterozygous, X-linked and de novo variants, and retain splice site, stop gain/loss, start gain/loss, frameshift, inframe insertion/deletion, and missense variants, as well as variants in 5’ and 3’ UTRs, as annotated by Ensembl’s Variant Effect Predictor tool [30]. This retained a total of 980 variants. These remaining variants were prioritized based on their likelihood of having a highly deleterious functional consequence based on the gene’s pLI score [31] and in silico predictions by SIFT [32], PolyPhen2 [33] and CAROL [34] functional pathogenicity prediction algorithms, previously established associations with phenotypes or disorders, relevant biochemical/biological function, and fetal or early postnatal brain-enriched spatiotemporal expression [7].

### Sanger sequencing

Variants identified in *TCF20* by WES were validated by PCR followed by Sanger sequencing.

Primers were designed using Primer-BLAST [35]. For II:1, the forward primer 5’ TGTACAAACGGCTCCAAGTTC 3’ and the reverse primer 5’ AGTGACGATTTCACCGAAGC 3’ were used to generate a 391 bp product encompassing the variant NC_000022.11:g.42210575del.

All PCR products were generated using the Expand High Fidelity PCR System (Roche) with the following thermocycler conditions: 5 min initial denaturation at 94 °C, with 30 cycles of 30 s denaturation at 94 °C, 30 s of annealing at 55 °C, and 45 s of extension at 72 °C, followed by 7 min of final extension at 72 °C.

Sanger sequencing was performed by Auckland Genomics, The University of Auckland, New Zealand. Electropherograms were viewed using Geneious (v8.1.5) (http://www.geneious.com).

Confirmation of the variant in II:2 was performed by All Wales Medical Genetics Service (United Kingdom) using PCR and Sanger sequencing. Both parents were also tested for the variant.

### Barcoded Illumina MiSeq sequencing

In an attempt to detect the presence of the variant in low level mosaic form in the parents, the genomic *TCF20* region described above was amplified from peripheral whole blood of each parent.

Targeted amplification was performed using PCR with the PCR primers previously described, adapted to include the Illumina overhang adapter sequences (underlined); forward primer (5’ TCGTCGGCAGCGTCAGATGTGTATAAGAGACAGTGTACAAACGGCTCCAAGTTC 3’) and reverse primer (5’ GTCTCGTGGGCTCGGAGATGTGTATAAGAGACAGAGTGACGATTTCACCGAAGC 3’).

Each PCR was performed using the Expand High Fidelity PCR System (Roche), with the following conditions: 5 min initial denaturation at 94 °C, with 30 cycles of 30 s denaturation at 94 °C, 30 s of annealing at 55 °C, and 45 s of extension at 72 °C, followed by 7 min of final extension at 72 °C. Amplified product was purified using AMPure XP beads (Beckman Coulter). The purified DNA was quantified using Qubit 2.0 fluorometer (Invitrogen) via the Qubit dsDNA Broad Range Assay Kit. MiSeq sequencing was performed by Auckland Genomics, The University of Auckland, New Zealand. Sequence reads were aligned to the target reference (GRCh38/hg38) using Burrow-Wheeler Aligner (BWA-MEM) [36] and visualized using the Integrated Genomics Viewer (IGV, v2.3.55) [28].

### MinION deep sequencing

To further probe the presence of the variant in rare form (e.g. less than 10% allele fraction) in the parents’ blood, we performed MinION sequencing (Oxford Nanopore Technologies) of PCR amplicons from parental peripheral whole blood DNA.

Targeted *TCF20* amplification was performed using the primers and conditions described for Sanger sequencing. Amplified product was purified using AMPure XP beads (Beckman Coulter). The purified DNA was quantified using Qubit 2.0 fluorometer (Invitrogen) via the Qubit dsDNA Broad Range Assay Kit.

The library was prepared for Oxford Nanopore Technologies’ (ONT) MinION sequencing as in the 1D Native barcoding DNA (with EXP-NBD103 and SQK-LSK108) protocol (ONT). The MinION sequencer was run using a single new R9.4 flowcell. Base calling was performed using guppy basecaller v2.3.7 (ONT) from the raw fast5 sequencing files from the MinION sequencer, followed by barcode demultiplexing also using guppy barcoder v2.3.7 (ONT), generating a fastq file for each barcode. Alignment of barcoded reads to the TCF20 target region was performed using minimap2 v2.16 (using the GRCh37/hg19 reference genome) and visualized using the Integrated Genomics Viewer (IGV, v2.3.55) [28].

### Droplet digital PCR and fluorescence

To assess parental mosaicism in cells derived from a different germ layer, droplet digital PCR was performed on DNA from urine (Canterbury Health Labs, Christchurch, New Zealand). The *TCF2*0 familial variant was amplified by PCR in the presence of a fluorescent interchelating dye, EvaGreen (BioRad), using the manufacturer’s recommended conditions. The wildtype forward primer 5’ GAGAGCCAAAGCCAAAAAAACAGAGGCAAAGG 3’, variant forward primer 5’ GAGAGCCAAAGCCAAAAAACAGAGGCAAAGG 3’ and the reverse primer 5’ TTTGATCTCAGGTTCTTGGGGTTCCACA 3’ were used to generate a 122 bp amplicon. Droplet fluorescence was then measured on a QX200 Droplet Digital PCR system and data was analyzed using QuantaSoft software. Quantitation was carried out by normalizing the variant allele to the wildtype allele. In a known heterozygous control (II:1) this ratio was 50%.

## Results

### Variant identification

**Fig. 1:**
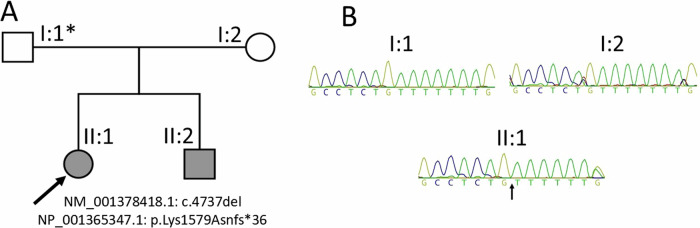
Summary of the TCF20 variant. **A** Pedigree showing the immediate family of the proband (II:1, indicated by the arrow) and affected sibling (II:2). The germline mosaic genotype of the father of the affected siblings is marked with an asterisk. **B** Sanger sequencing chromatograms of peripheral blood-derived DNA of the proband and her parents, with the arrow pointing to the thymidine deletion in the proband

The sole variant in II:1 (case 81 in Musgrave et al. [37]) retained after filtering was a heterozygous single nucleotide deletion of a thymidine in exon 1 (of 6) of *TCF20*, NC_000022.11:g.42210575del; NM_001378418.1:c.4737del; NP_001365347.1:p.Lys1579Asnfs*36 (GRCh38/hg38; MANE Select transcript), Class 5 Pathogenic (PVS1, PM2_Supp, PS2_mod) [38] – Fig. 1. The variation lies within a 7-nucleotide thymidine homopolymer tract. This variant was subsequently confirmed to be present in sibling II:2, but was not detectable in electropherograms obtained from Sanger sequencing of the parents.

### Assessment of parental mosaicism

To investigate potential parental mosaicism, deep sequencing of amplicons generated from DNA extracted from peripheral blood encompassing the variant in both parents and the proband was performed by Illumina MiSeq short-read sequencing. Read depth at the variant locus exceeded 105,000 in every sample. The deletion was present in 50,825/105,340 (48.2%) of reads for II:1, confirming heterozygosity at this locus. The single thymidine deletion was present in 2036/150,247 (1.36%) and 1851/233,680 (0.79%) of the reads for the mother I:2 and father I:1, respectively.

Next, potential parental mosaicism was also investigated using ONT MinION sequencing for the amplicon encompassing the variant in both parents and proband also in DNA extracted from peripheral blood. The read depth for each individual exceeded 15,000 reads. Alignments for all three individuals showed over 50% of reads with a 6-nucleotide long thymidine homopolymer. Additionally, >20% of aligned reads contained a 5-nucleotide long thymidine homopolymer.

**Fig. 2:**
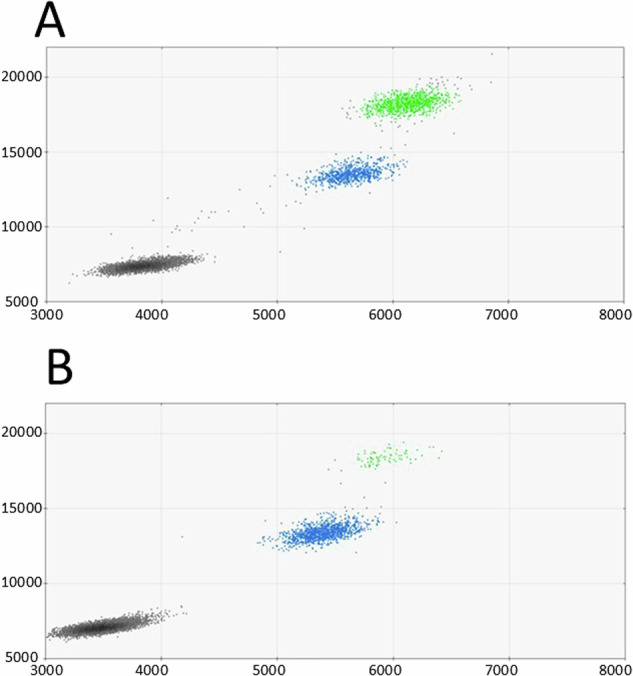
Two-dimensional ddPCR scatter plots for the analysis of the TCF20 variant. **A** Proband’s ddPCR scatter plot from peripheral blood. **B** Proband’s father’s ddPCR scatter plot from urothelial cells. Colored dots represent single droplets of emulsion carrying amplified DNA harboring the variant (green) or wildtype DNA (blue). Black dots represent droplets with no amplification of the target DNA

Finally, parental mosaicism was investigated using ddPCR from DNA extracted from urothelial cells from early morning urine from the parents. First morning urine was chosen as a DNA source for ddPCR, to have a population of cells that more closely resembles the original gonadal tissue (compared to white blood cells). Interestingly, the variant was detected by ddPCR in 3.2% of paternal DNA (Fig. 2) and was absent in the maternal sample.

## Discussion

### *TCF20* variant and predicted consequence

**Fig. 3:**
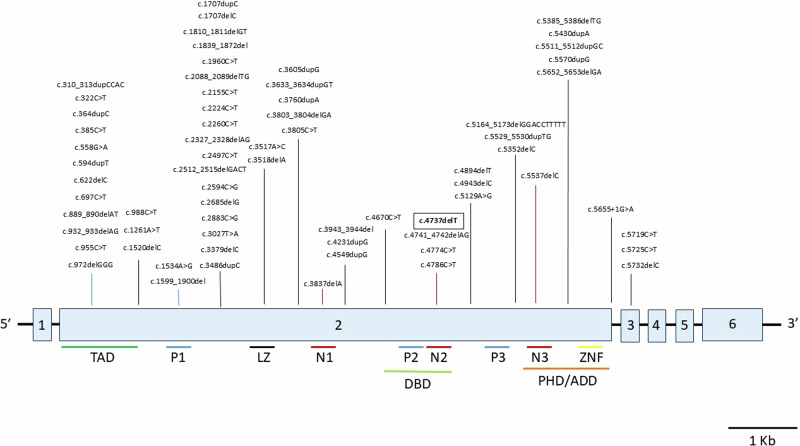
Previously reported TCF20 variants. All SNVs and indels (76 total) in *TCF20* are represented. Structural and copy number variants are not shown (see Supplementary Table 1 for a full list of previously reported copy number and structural variants). The variant from this report is framed in bold. Exons are indicated in light blue and numbered. Protein domains are detailed under the exon diagram. Abbreviations: TAD = transactivation domain; P1/2/3 = PEST domains 1/2/3; LZ = leucine zipper; N1/2/3 = nuclear localization signals 1/2/3; DBD = DNA-binding domain; PHD/ADD = Plant Homeodomain/ADD; ZNF = zinc finger domain

WES and Sanger sequencing analysis revealed a previously unreported single base pair heterozygous deletion, NM_001378418.1:c.4737del; NP_001365347.1:p.Lys1579Asnfs*36 (GRCh38/hg38), in the proband (II:1, Fig. 1) and her brother (II:2) located within the region encoding the N2 domain of the TCF20 protein amidst a stretch of four other previously reported neurodevelopmental disorder-associated variants (spanning c.4737 to c.4786; Fig. 3), reported as pathogenic in ClinVar (NCBI) (accession number VCV002571818.3). The genetic variant deletes a thymidine in a homopolymer tract of seven thymidine residues (22:g.42210568-42210575), inducing a frameshift resulting in the replacement of the C-terminal 381 amino acid residues with 34 residues not found in the wild-type protein. The frameshift replaces 21 residues of the 25 amino-acids long second nuclear localization signal (N2) [39], potentially affecting the ability of the truncated TCF20 protein to localize to the nucleus to exert its transcription factor binding activity. As the variant is located in the second of six exons, degradation of the *TCF20* mRNA harboring the variant via nonsense mediated decay is another potential consequence.

Given that *TCF20* is highly intolerant to loss of function variants, with a pLI of 1.0 [31], its ranking in the top 23.45% of genes most intolerant to haploinsufficiency [40], and the overlap of the symptoms observed in both siblings with previous *TCF20*-associated disorders (see Supplementary Table 1 for details), the variant was considered causative of the siblings’ phenotype.

### *TCF20*-associated neurodevelopmental disorders

Including the siblings reported here, a total of 76 unique disorder-causing variants in *TCF20* have now been reported across 91 individuals from 83 different families to date (see Supplementary Table 1 for details) [3, 14, 16–24, 26, 41]. Causative variants in *TCF20* include SNVs (36.3%), indels (50.5%), copy number variants (CNVs) (9.9%), and structural variants (SVs) (3.3%). Of the 87 individuals in whom gender was reported, these *TCF20* variants affected 32 females (36.8%) and 55 males (63.2%). Most individuals have developmental/intellectual disability (present in 85 out of 91 cases, i.e. 93.4% of cases), autism (present in 48 out of 86 reported cases, i.e. 55.8% of cases), and dystonia [22]. However, dysmorphisms, neurological findings, hepatic issues [23], and other organ system-specific impairments appear to be variable among cases (see Supplementary Table 1 for details), highlighting the morphological heterogeneity of *TCF20*-associated neurodevelopmental disorder.

### Germline mosaicism in *TCF20*

As the MiSeq error rates for homopolymer runs are equivalent to the proportion of reads with a single T deletion in the parents [42], we initially concluded there was no definitive indication of mosaicism in either parent using this approach in DNA extracted from blood. On MinION sequencing, the alignments were indistinguishable between the proband and two parents: Alignments from all three individuals showed over 50% of reads with a 6-nucleotide long thymine homopolymer, and 20% of aligned reads contained a 5-nucleotide long thymine homopolymer, highlighting the difficulty of accurately determining the count of residues in homopolymer sequences. The variant was, however, confidently detected by droplet digital PCR in 3.2% of paternal DNA extracted from urine (urothelial cells), confirming germline inheritance.

There are now four independent reports of either suspected (affected siblings carrying the same variant not detectable in parents) or confirmed germline mosaic variants in seven individuals with *TCF20*-associated disorders from four different families. The cases with germline mosaicism consist of 1) a pericentric chromosome 22 inversion affecting both the *TCF20* and *ACTN6B* genes resulting in intellectual disability and autism in brothers not detected in parental blood/lymphoblastoid cell-derived DNA [16], 2) a substitution (NM_005650.1:c.2224 C > T) in siblings with autism not identified in parental blood-derived DNA [26], 3) a substitution (NM_005650.1:c.558 G > A) in a male with intellectual disability and autism, found in low-level germline mosaic form (6.98%) in his mother’s peripheral whole blood [21], and 4) in this report, a thymidine deletion (NM_001378418.1:c.4737GT>G) in siblings with intellectual disability and autism identified in low-level germline mosaic form in the father’s urine. These are in addition to 67.0% (61 out of 91) of cases in which *TCF20* variants have arisen apparently de novo (see Table 1), which could be cases of undetected germline mosaic inheritance from a parent.

**Table 1:** Variant types and modes of inheritance of disorder-associated *TCF20* variants, including those previously reported and those from our case report

	De novo	Inherited (maternally)	Inherited (paternally)	Germline mosaic (suspected or confirmed)	Undetermined	TOTAL	Fraction of all variants (%)
SNV	20	2	3	3	5	33	36,3
Indel	32	2	2	2	8	46	50,5
CNV	8	0	0	0	1	9	9,9
Other SV	1	0	0	2	0	3	3,3
Total number of variants	61	4	5	7	14	91	
Fraction of all variants (%)	67,0	4,4	5,5	7,7	15,4		

Interestingly, Amiel et al. [43] found that 12 of 20 cases with *PAX2*-associated renal-coloboma syndrome were caused by either an insertion or a deletion of a single residue from a stretch of homopolymer of seven guanines. This may indicate the presence of a variant hotspot linked to strand slippage during replication, including in one familial case of suspected paternal germline mosaicism. A similar mechanism could be at play in the *TCF20* thymidine tract.

### Germline mosaicism proposed as a recurring mode of inheritance in neurodevelopmental disorders

Since germline mosaicism was first identified in studies of the mutagenic effects of mustard gas [44], it has been invoked to explain the presence of recurrent rare dominant disorders in children of unaffected parents [45–47]. More recently, attention has focused increasingly on the genetic architecture of neurodevelopmental disorders which appear to be highly enriched in de novo variants in key brain developmental genes [48–53], including in germline mosaic form [54]. Conditions with germline mosaicism as a recurrent mode of inheritance include Rett syndrome [55], Fragile X syndrome [56], and tuberous sclerosis complex (TSC) [57]. A recent study using a single-molecule Molecular Inversion Probe (smMIP) enrichment method followed by parental blood DNA sequencing at a depth of ~7000 found that nearly 4% of all cases of reported de novo variants in individuals affected by a range of NDDs were transmitted from mosaic parents, a proportion which is even higher in epileptic individuals with NDDs [58].

In addition, a number of case reports for a range of conditions have shown that variants which were initially thought to have arisen de novo were inherited from a parent with low-level (<10% in allele fractions, as defined in [59, 60] germline mosaicism [61–63]. In a study by Xu et al., approximately 10% of variants in the sodium channel gene *SCN1A* (which cause Dravet syndrome), initially considered de novo, were identified by deep amplicon resequencing to have resulted from either maternal or paternal mosaicism at allele frequencies ranging from 1.1 to 32.6% [62]. Consistently, Zillhardt et al. found that both maternal and paternal germline mosaicism accounts for up to 15% of recurrent malformations of cortical development [64], while Breuss et al. on analyzing sperm DNA from fathers of children with autism, revealed that many harbored germline mosaic autism-associated variants, with allele fractions ranging from 2 to 15% [63].

Furthermore, the inheritance of low-level germline mosaic variants is not limited to pathogenic variants, as many germline mosaic variants have been identified in healthy families with no associated clinical phenotypes. Rahbari et al. assessed rates of de novo SNVs in unaffected multi-sibling families to find that ~3.8% of these were in fact result of parental germline mosaicism, at alternate allele fractions ranging from 0.57% to 10.24%, with roughly half of maternal and half of paternal origin. In parallel, 1.3% of de novo variants were found to be shared among siblings, reaching 24% for de novo variants which were mosaic in >1% of parental blood-derived cells, and 50% for de novo variants that were present in >6% of parental blood-derived cells [65]. Another large study conducted by Jónsson et al. found that 57.2% of de novo variants (including SNVs and indels) shared by siblings were confirmed to be found in mosaic form in parental blood [47].

The selfish spermatogonial selection hypothesis suggests a mechanism to explain the prevalence of such pathogenic variants in the male germline. It highlights that a sperm cell that has acquired a variant that confers a selective advantage, in quantity (by clonal expansion) and/or quality (by increased motility and survivability), is more likely to survive and fertilize, even if this variant eventually leads to a disorder over the course of development [66–70]. This is of particular salience to the context of neurodevelopmental disorders since variants which enhance processes of cellular hyperproliferation and motility will, when brain-expressed, amplify the very mechanisms by which certain disorders of brain development, such as autism, macrocephaly and RASopathies, arise or are exacerbated [71]. Interestingly, this spermatogonial selection hypothesis appears to provide a parsimonious explanation for the correlation between de novo variants and advanced paternal age [68, 72].

### Limitations in the detection of germline mosaicism

The likely under-detection of germline mosaicism could be a consequence of both limitations with biological sampling and DNA sequencing technology.

First, if only one sibling inherits a variant, germline mosaicism in a parent will not be conspicuously suspected. Therefore, cases of germline mosaicism in which only one sibling is affected are likely to go unnoticed [73].

Second, either paternal or maternal germline mosaicism is difficult to identify if the variant exclusively affects cell types not included in the sequenced sample. In our study for example, the identification of the *TCF20* variant in a proportion of urothelial cells raises the possibility that the variant arose in the father early on in embryonic development, presumably affecting cells giving rise to sperm and urothelial cells, but not blood cells, a process described by Rhabari et al. [65]. This is consistent with a study showing that only about one third of sperm mosaic variants are detectable in blood cells [74], and data demonstrating that the analysis of the fathers’ blood alone may underestimate the presence of germline mosaicism in fathers of children with intellectual disability syndromes [75].

Finally, even at very high read depths (e.g. 100,000x), limitations inherent to sequencing target preparation and sequencing technologies limit their ability to detect certain types of variants, irrespective of the allele frequency. First, Illumina MiSeq sequencing has a published error rate of 1.85–2.5% when sequencing homopolymer runs, specifically, 1.85% for guanine or cytosine 6- to 7 bp homopolymer runs [76], and ~2.5% for thymidine or adenine 7-bp homopolymer runs [42]. These previously reported error rates for homopolymer runs are equivalent to the proportion of reads with a single T deletion observed in the MiSeq results for both parents, which could, in our case, have been misinterpreted as the presence of the *TCF20* variant in the parents. Second, similar to Illumina MiSeq sequencing, the difficulty of basecalling ONT sequence-surrounding stretches of low complexity is a well-reported limitation of the technology [77–80]. For the flowcells used in this experiment (R9.4), the base composition in the pore is primarily determined from the three central nucleotides located within the pore. Thus, accurately determining the length of a homopolymer tract greater than four nucleotides (including the 7 nucleotide thymidine homopolymer seen in the proband) is technically challenging [81]. In our case this meant that neither MiSeq sequencing at 105,000 read depth nor MinION Oxford Nanopore sequencing at >15,000 read depth of targeted PCR products were able to reliably detect the proband’s deletion [82, 83].

Considering the limitations linked to the complexity of hierarchical multicellular organism development—and the need for tissue source and sequencing technology to match a variant’s distribution, nature, and allele fraction—germline mosaic variants may remain under-detected in *TCF20*-associated neurodevelopmental disorders and neurodevelopmental conditions more generally. In the future, it will be interesting to profile the distribution of different types of genetic variants that arise in germline mosaic form, investigate any mutagenic mechanistic links to environmental exposures [84], and understand how they might differentially affect coding versus noncoding genomic regions.

## Conclusion

In conclusion, we here broaden the spectrum of genetic variation, provide the second known case of confirmed germline mosaic *TCF20*-associated disorders and first known case of confirmed germline mosaic inheritance of the same *TCF20* variant in siblings. In light of a clearly growing subset of patients with a positive family history of *TCF20*-associated neurodevelopmental disorders [21], our report demonstrates that parental testing of individuals with *TCF20*-associated neurodevelopmental disorders is critical, and that special attention should be paid to capturing low-level mosaicism in order to provide the most accurate genetic counselling and recurrence risk assessments possible. Germline mosaic variants may represent a clinically salient yet under-detected mode of disorder-associated inheritance not only in *TCF20*-associated disorder, but other neurodevelopmental disorders as well [85–87].

## Supplementary information

Supplementary Table 1

Supplementary Table 1 - descriptive summary
