# Supplementary material for: Germline mosaicism in TCF20-associated neurodevelopmental disorders: a case study and literature review
Source: J Hum Genet. 2025 Feb 26;70(4):215–22. doi: 10.1038/s10038-025-01323-3 (PMC11882450; doi:10.1038/s10038-025-01323-3)
Supplement: Supplementary file 2 — Supplementary Table 1 - descriptive summary [file 10038_2025_1323_MOESM2_ESM.docx]

**Germline mosaicism in *TCF20*-associated neurodevelopmental disorders: a case study and literature review**

**Supplementary information description**

The supplementary table (Supplementary Table 1) (.xls format) includes a summary of all reported neurodevelopmental disorder-associated *TCF20* variants from previously published cases, as referenced, and an analysis of variant types (SNV; indel; CNV; other SV) and modes of inheritance (de novo; maternally inherited; paternally inherited, germline mosaic, likely or confirmed) across all cases.
